# Supplementary material for: Association between mean platelet volume and obstructive sleep apnea-hypopnea syndrome: A systemic review and meta-analysis
Source: PLoS One. 2024 Feb 16;19(2):e0297815. doi: 10.1371/journal.pone.0297815 (PMC10871486; doi:10.1371/journal.pone.0297815)
Supplement: S1 Table — (DOCX) [file pone.0297815.s001.docx]

**Supplemental Material**

**S1 Table.** The exact search terms of each database

| **Database** | **Search strategies** | **Results** |
| --- | --- | --- |
| **PubMed** | #1 ("Mean Platelet Volume"[Mesh]) OR (mean platelet volume[Title/Abstract] OR MPV[Title/Abstract]) ---5,871  #2 ("Sleep Apnea Syndromes"[Mesh]) OR (sleep apnea syndrome[Title/Abstract] OR sleep apnea[Title/Abstract] OR sleep apnea syndrome[Title/Abstract] OR sleep hypopnea[Title/Abstract] OR mixed central[Title/Abstract] AND obstructive sleep apnea[Title/Abstract] OR mixed sleep apnea[Title/Abstract] OR hypersomnia with peri odic respiration[Title/Abstract] OR obstructive sleep apnea hypopnea syndrome[Title/Abstract] OR OSAHS[Title/Abstract] OR OSA[Title/Abstract] OR OSAS[Title/Abstract])---54,806  #3 #1 AND #2---50 | 50 |
| **Web of**  **Science** | #1 ((TS=(mean platelet volume)) OR TI=(mean platelet volume OR MPV)) OR AB=(mean platelet volume OR MPV) ---6,533  #2 ((TS=(sleep apnea syndromes)) OR TI=(sleep apnea syndrome OR sleep apnea OR sleep apnea syndrome OR sleep hypopnea OR mixed central and obstructive sleep apnea OR mixed sleep apnea OR hypersomnia with peri odic respiration OR obstructive sleep apnea hypopnea syndrome OR OSAHS OR OSA OR OSAS)) ---44,813  #3 #1 AND #2---63 | 63 |
| **Embase** | #1 'mean platelet volume'/exp OR 'mean platelet volume':ti,ab,kw OR mpv:ti,ab,kw ---11,476  #2 'sleep apnea syndromes'/exp OR (('sleep apnea':ti,ab,kw OR 'sleep apnea syndrome':ti,ab,kw OR 'sleep hypopnea':ti,ab,kw OR 'mixed central':ti,ab,kw) AND 'obstructive sleep apnea':ti,ab,kw) OR 'mixed sleep apnea':ti,ab,kw OR 'hypersomnia with peri odic respiration':ti,ab,kw OR 'obstructive sleep apnea hypopnea syndrome':ti,ab,kw OR osahs:ti,ab,kw OR osa:ti,ab,kw OR osas:ti,ab,kw ---111,367  #3 #1 AND #2---48 | 48 |
| **CNKI** | #1 (SU=mean platelet volume) OR (TKA=mean platelet volume OR MPV) ---4,074  #2 (SU=sleep apnea syndromes) OR (TKA=sleep apnea syndrome OR sleep apnea OR sleep apnea syndrome OR sleep hypopnea OR mixed central and obstructive sleep apnea OR mixed sleep apnea OR hypersomnia with peri odic respiration OR obstructive sleep apnea hypopnea syndrome OR OSAHS OR OSA OR OSAS) ---7,370  #3 #1 AND #2---41 | 41 |
| **Wan Fang** | #1 theme words:(mean platelet volume) or title or keywords:(mean platelet volume OR MPV)---8,694  #2 theme words:(sleep apnea syndromes) or title or keywords:(sleep apnea syndrome OR sleep apnea OR sleep apnea syndrome OR sleep hypopnea OR mixed central and obstructive sleep apnea OR mixed sleep apnea OR hypersomnia with peri odic respiration OR obstructive sleep apnea hypopnea syndrome OR OSAHS OR OSA OR OSAS)---35,493  #3 #1 AND #2---38 | 38 |
