# Supplementary material for: Association between mean platelet volume and obstructive sleep apnea-hypopnea syndrome: A systemic review and meta-analysis
Source: PLoS One. 2024 Feb 16;19(2):e0297815. doi: 10.1371/journal.pone.0297815 (PMC10871486; doi:10.1371/journal.pone.0297815)
Supplement: S2 Table — (DOCX) [file pone.0297815.s002.docx]

library(meta)

m1

a<-metacor(cor = r,n = n,studlab = studlab,data=m1,sm="cor",backtransf = FALSE)

a

forest(a,comb.fixed = TRUE)

funnel(a)

metabias(a,method.bias = "linreg",plotit = T,k.min=6)

forest(metainf(a))

ranktest(a)

regtest(metamod)
