# Supplementary figures and images for: Association between mean platelet volume and obstructive sleep apnea-hypopnea syndrome: A systemic review and meta-analysis
Source: PLoS One. 2024 Feb 16;19(2):e0297815. doi: 10.1371/journal.pone.0297815 (PMC10871486; doi:10.1371/journal.pone.0297815)

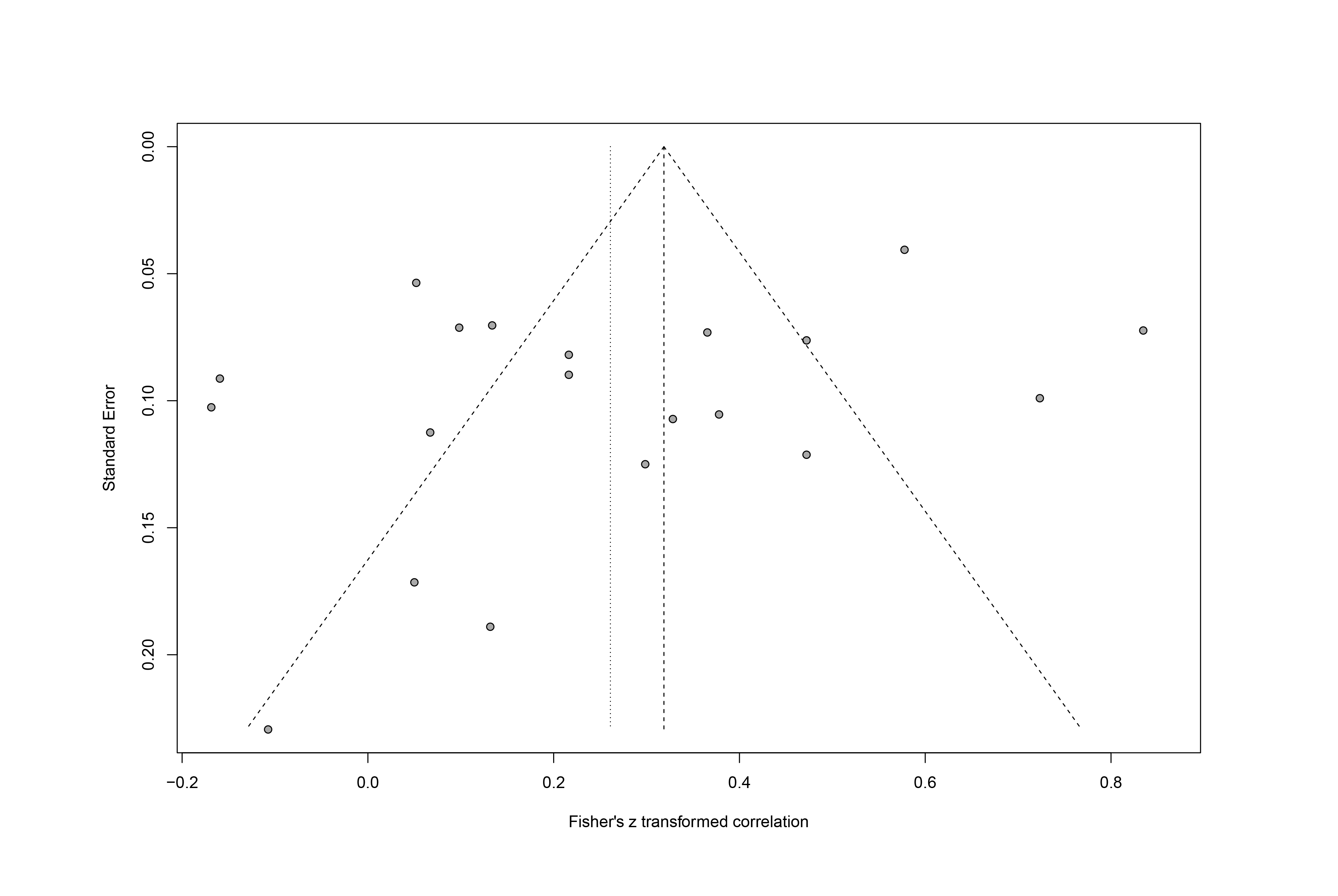

Supplement: S1 Fig — (TIF) [file pone.0297815.s003.tif]
